# Efficient Enrichment of Retinal DHA with Dietary Lysophosphatidylcholine-DHA: Potential Application for Retinopathies

**DOI:** 10.3390/nu12103114

**Published:** 2020-10-12

**Authors:** Dhavamani Sugasini, Poorna C. R. Yalagala, Papasani V. Subbaiah

**Affiliations:** 1 Department of Medicine, Section of Endocrinology and Metabolism, University of Illinois at Chicago, Chicago, IL 60612, USA; sugasini@uic.edu (D.S.); Yalagala@uic.edu (P.C.R.Y.); 2 Jesse Brown VA Medical Center, Chicago, IL 60612, USA

**Keywords:** Omega-3 fatty acids, retina, lysophospholipid, fish oil, docosahexaenoic acid (DHA), krill oil, eicosapentaenoic acid (EPA), Mfsd2a (major facilitator superfamily domain-containing protein 2a), blood–retina barrier

## Abstract

Although decreased retinal docosahexaenoic acid (DHA) is a known risk factor for retinopathy, currently available omega-3 fatty acid supplements, which are absorbed as triacylglycerol (TAG), do not significantly enrich retinal DHA. We tested the hypothesis that lysophospahtidylcholine (LPC)-DHA which is absorbed as phospholipid, would efficiently increase retinal DHA because of the presence of LPC-specific transporter at the blood–retina barrier. In normal rats, LPC-DHA and di-DHA phosphatidylcholine (PC), which generates LPC-DHA during digestion, increased retinal DHA by 101% and 45%, respectively, but TAG-DHA had no significant effect at the same dose (40 mg/kg, 30 days). In normal mice, both sn-1 DHA LPC and sn-2 DHA LPC increased retinal DHA by 80%, but free DHA had no effect. Lipase-treated krill oil (which contains LPC-DHA and LPC-EPA (eicosapentaenoic acid), but not normal krill oil (which has little LPC), increased both retinal DHA (+76%) and EPA (100-fold). Fish oil, however, had no effect, whether lipase-treated or not. These studies show that retinal DHA can be efficiently increased by dietary LPC-DHA, but not by TAG-DHA or free DHA. Since DHA is known to be protective against retinopathy and other eye diseases, this study provides a novel nutraceutical approach for the prevention/treatment of these diseases.

## 1. Introduction

Of all the tissues in the body, retina contains the highest concentration of the omega-3 fatty acid (FA), docosahexaenoic acid (DHA). Up to 60% of the total FA in rod outer segment membrane phospholipids is DHA [1]. In addition, retina uniquely contains significant amounts of dipolyenoic phospholipids with very long chain (up to 36 carbons) omega-3 polyunsaturated FA (VLCFA) at sn-1 position, and DHA at sn-2 position [2]. This unusual FA composition of retina is believed to have physiological significance for the photoreceptor function. Retina is almost completely dependent upon dietary supply of DHA since it cannot synthesize DHA from the linolenic acid (18:3, n-3) precursor [2]. Several epidemiologic [3,4] and pre-clinical [5,6] studies have shown that dietary omega-3 FA protect against retinal diseases, whereas DHA deficiency is associated with impaired visual function [3]. Moreover, retinal DHA is significantly reduced in diabetes [6,7,8], retinitis pigmentosa [9,10], age-related macular degeneration [4], and peroxisomal disorders [11], and this deficiency appears directly correlated with functional defects, including impaired visual development and reduced sensitivity to light [12]. Despite evidence from the epidemiologic and animal studies for the beneficial effects of omega-3 FA for retinal function, controlled clinical trials with currently available supplements in patients with diabetic retinopathy [2,13], and age-related macular degeneration [14] have shown no appreciable benefits. We postulate that the reason for this failure is the inability of the currently available supplements (including fish oil, ethyl esters, free fatty acids, krill oil, algal oil) to significantly enrich retinal DHA at clinically feasible doses. This is because they are all absorbed predominantly in the form of triacylglycerol (TAG) [15,16], whereas enrichment of brain and retina requires a phospholipid form of DHA [17]. Although previous studies have shown that the uptake of DHA by retina involves lipoprotein receptors [3], adiponectin receptor [18], and multiple fatty acid transporters [19], more recent studies suggest that the predominant pathway for DHA uptake by retina may be through the Mfsd2a (major facilitator superfamily domain-containing protein 2a) pathway [20]. Since the DHA from the currently used supplements is absorbed mostly as TAG, whereas the Mfsd2a pathway requires a lysophospholipid form of DHA [17], it is possible that retinal DHA was not appreciably enriched by these supplements. We have recently demonstrated that if the dietary DHA is provided as LPC, it efficiently enriches brain DHA in adult mice and rats, and also improves their brain function [15,16,21]. Since the uptake of DHA by retina and brain appear to be similar [20], we tested the hypothesis that the retinal DHA also can be increased by dietary lysophospahtidylcholine (LPC)-DHA. The results presented here show that in both adult mice and rats, the retinal DHA is significantly increased by low dose dietary LPC-DHA, whereas free DHA or TAG-DHA at similar dose do not show significant effect. These results are potentially significant in the prevention and clinical management of retinal diseases through diet.

## 2. Materials and Methods

Animals and dietary treatments: All studies in animals described here were approved by the Institutional Animal Care and Use Committee of the University of Illinois at Chicago. The retina samples were obtained from our previous studies on the brain accretion of DHA in mice and rats, which were published previously [15,16,21]. Male Sprague-Dawley rats (8-week-old) were purchased from Harlan laboratories (Indianapolis, IN, USA). Male C57 BL/J6 mice (2–4-months-old) were purchased from Jackson Laboratories (Bar Harbor. Maine).

In the first study, male Sprague-Dawley rats (*n* = 5 per group, 8-week-old) were gavaged daily with 10 mg of DHA (40 mg DHA/kg body weight) in the form of TAG-DHA (DHASCO algal oil, DSM Nutritional Products, Columbia, MD, USA), synthetic di-DHA PC (phosphatidylcholine), or synthetic LPC-DHA (sn-1 acyl) for 30 days [15]. The DHA was distributed equally among the three positions of TAG-DHA [15]. In this study we have also included another group of rats which were gavaged with a half dose of LPC-DHA (20 mg DHA/kg) to be comparable with the expected amount of LPC-DHA generated from di-DHA PC during digestion. In the second study, 4-month-old male C57 BL/J6 mice (*n* = 8 per group) were gavaged daily with 40 mg DHA/kg body weight in the form of free (unesterified) DHA, sn-1 acyl LPC-DHA, or sn-2 acyl LPC-DHA for 30 days as described previously [16]. In the third study, male C57 BL/J6 mice (2-months-old, *n* = 5 per group) were fed diets enriched with natural or lipase-treated fish oil or krill oil for 30 days. The total amount of omega-3 FA (eicosapentaenoic acid (EPA)+DHA) was 2.64 g/kg diet in all the diets. The untreated krill oil contained 18% of total omega-3 FA as LPC, whereas the lipase-treated krill oil contained >80% of the total omega-3 FA as LPC. The fish oil diets contained no LPC-EPA or LPC-DHA. The animals were trans-cardially perfused with ice cold phosphate buffered saline under anesthesia and the retinas were collected and kept frozen at −80 °C until the analysis.

Analytical procedures: The total lipids of retina were extracted by Bligh and Dyer procedure [22] and the fatty acids were methylated using methanolic HCl. The fatty acid analysis was carried out by GC/MS (gas chromatography/mass spectroscopy) using Shimadzu QP2010SE equipped with Supelco Omegawax column, as described previously [16]. Total ion current in the range of 50–400 m/z was used for quantification of the methyl esters. For LC/MS/MS (liquid chromatography/tandem mass spectroscopy) analysis, the lipids were extracted by the procedure of Ivanova et al. [23]. The analysis of molecular species of phospholipids was performed on an ABSciex QTRAP mass spectrometer (Redwood City, CA, USA) coupled with Agilent 2600 UPLC system (Santa Clara, CA, USA), by multiple reaction monitoring [24]. The internal standards 17:0 LPC, di 17:0 PC, and di 17:0 PE (phosphatidylethanolamine) were used for the quantification of the corresponding molecular species, without applying any correction factors for the differences in the ion intensity of different molecular species.

Statistics and correlations: The significance of differences between treatment groups was determined by a one-way ANOVA, with Tukey post-hoc multiple comparison corrections or unpaired *t*-test between control and treated samples adjusted with Holm–Sidak method (Graphpad Prism 8.0, San Diego, CA, USA).

## 3. Results

### 3.1. Comparative Effects of Dietary DHA in the Form of PC, TAG, and LPC in Rats

**Figure 1:**
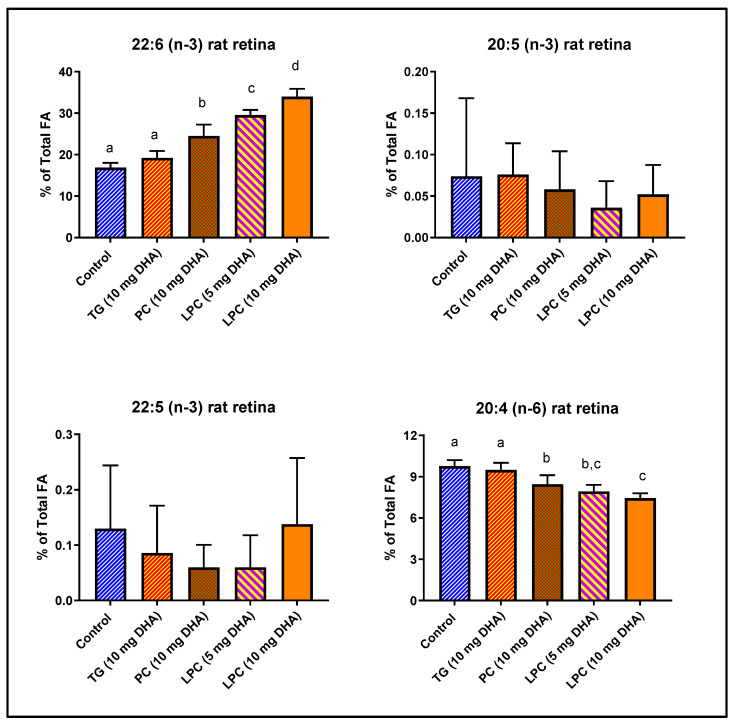
Incorporation of dietary DHA fed in the form of PC, TAG, or LPC into rat retinal lipids. Normal male Sprague-Dawley rats (8-week-old; *n* = 5 per group) were gavaged daily with 10 mg DHA in 250 µL corn oil in the form of TAG-DHA, di-DHA PC, or LPC-DHA for 30 days. In addition, a half-dose of LPC-DHA (5 mg DHA) was used to be comparable to the amount of LPC-DHA expected to be generated by di-DHA PC during digestion. All animals were on regular rodent chow that contained no DHA, but contained 17.4 mg α-linolenic acid (18:3, n-3) per g of chow. Retinal FA were analyzed by GC/MS as described in the text. The percent composition of 20:4 (n-6), 20:5 (n-3), 22:5 (n-3), and 22:6 (n-3) are shown here. The total FA composition is shown in Table 1. The significance of differences between the treatment groups was determined by a one-way ANOVA, with Tukey multiple post-hoc test (Graphpad Prism 8.0). Bars without common superscripts are significantly different from each other (*p* < 0.05). TG: triacylglycerol; PC: phosphatidylcholine; LPC: lysophosphatidylcholine; FA: fatty acid; GC/MS: gas chromatography/mass spectroscopy.

We have previously demonstrated that rat brain DHA is efficiently enriched by dietary LPC-DHA and di-DHA PC, but not by TAG-DHA [15]. Since the mechanism of DHA uptake appears to be similar for brain and retina [20], we determined the enrichment of retinal DHA in the same groups of animals. Normal rats were gavaged with 10 mg DHA/day (40 mg DHA/kg) in the form of TAG-DHA, di-DHA PC, or LPC-DHA (sn-1 acyl) for 30 days, and the retinal FA composition was determined by GC/MS. In addition, a half dose of LPC-DHA (20 mg/kg) was used, in order to be equivalent to the expected generation of LPC-DHA by the digestion of di-DHA PC in the intestine by pancreatic phospholipase A_2_ (PLA_2_). The concentrations of omega-3 FA and arachidonic acid are shown in Figure 1, while the total FA composition is shown in Table 1. The percentage of DHA in retina was significantly increased by di-DHA PC (+45%) and by LPC-DHA (+101%), but not by TAG-DHA (+13%, not significant). The half dose of LPC-DHA (5 mg/rat) was more efficient (+75%) than the full dose of di-DHA PC (+45%), indicating that the hydrolysis of di-DHA PC by the pancreatic PLA_2_ (phospholipase A_2_) may not be efficient. Remarkably, although the DHA content of normal rat retina is very high (16.88% of total FA), it was doubled by feeding the full dose of LPC-DHA (to 33.98% of total FA), indicating the wide range of retinal membrane DHA content that can be achieved through diet. The increase in DHA occurred largely at the expense of arachidonic acid as we found in the brain [15], but the DHA also replaced saturated FA (16:0 and 18:0) in the retina (Table 1). There were no changes in either EPA (20:5 (n-3)) or DPA (22:5 (n-3)) of retina by any of the treatments.

### 3.2. Comparative Effects of Free DHA and Isomers of LPC-DHA in Mice

**Figure 2:**
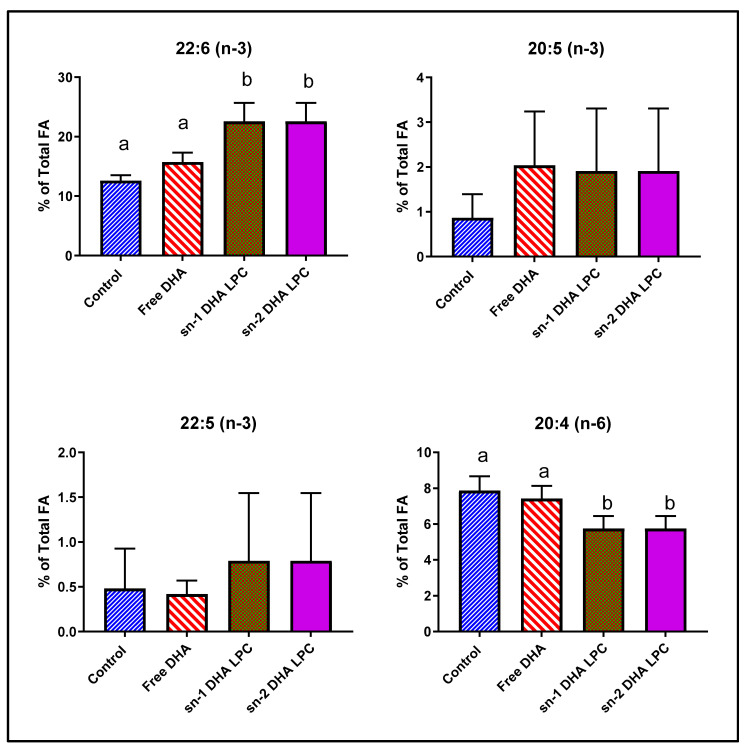
Effect of dietary free (unesterified) DHA, sn-1 DHA-LPC, and sn-2 DHA LPC on mouse retinal FA. Normal male mice (C57 BL/J6, 16-week-old) were gavaged daily with 1 mg DHA in the form of free DHA, sn-1 DHA LPC, or sn-2 DHA LPC in 80 µL corn oil for 30 days. Retinal FA composition was analyzed by GC/MS. Only the values for arachidonic acid and the omega-3 FA are shown here (mean ± SD, 8 animals/group). The total FA composition is shown in Table 2. Bars without common superscripts are significantly different from each other (*p* < 0.05) by a one-way ANOVA with Tukey post-hoc correction. SD: standard deviation; sn-1 and sn-2: stereospecific numbering 1 and 2 respectively; LPC: lysophosphatidylcholine; DHA: docosahexaenoic acid.

Previous studies suggested that for the uptake of DHA by the brain, the DHA has to be in the sn-2 position of LPC, since that is the natural position in phospholipids in vivo [25,26]. However, our recent studies showed that sn-1 DHA LPC and sn-2 DHA LPC were equally effective in enriching mouse brain DHA and in improving brain function [16]. We analyzed the retinal FA composition in mice fed free (unesterified) DHA, sn-1 DHA LPC, and sn-2 DHA LPC, to determine whether there is a preference for the sn-2 acyl isomer of LPC for transport through blood–retina barrier. As shown in Figure 2 and Table 2, free DHA had no effect on retinal DHA, similar to the effect of TAG-DHA in rats. However, both isomers of LPC-DHA increased the retinal DHA by 80%, showing that the uptake of DHA by retina is similar to that of brain, and involves the Mfsd2a transporter as shown by others [20]. Furthermore, the transporter at the blood–retina barrier did not distinguish between the two isomers of LPC-DHA, as we found for the brain [16]. There was no effect on either EPA (20:5, n-3) or DPA (22:5, n-3) content by any of the DHA treatments. There was a significant decrease in arachidonate by the two isomers of LPC-DHA, but not by free DHA. In addition, there were significant decreases in saturated fatty acids (16:0 and 18:0) as well as in 18:1 by LPC-DHA, but not by free DHA. These results suggest that, similar to the results in rats, the increase in mouse retinal DHA occurred by the replacement of not only arachidonate but also the saturated FA and 18:1.

**Figure 3:**
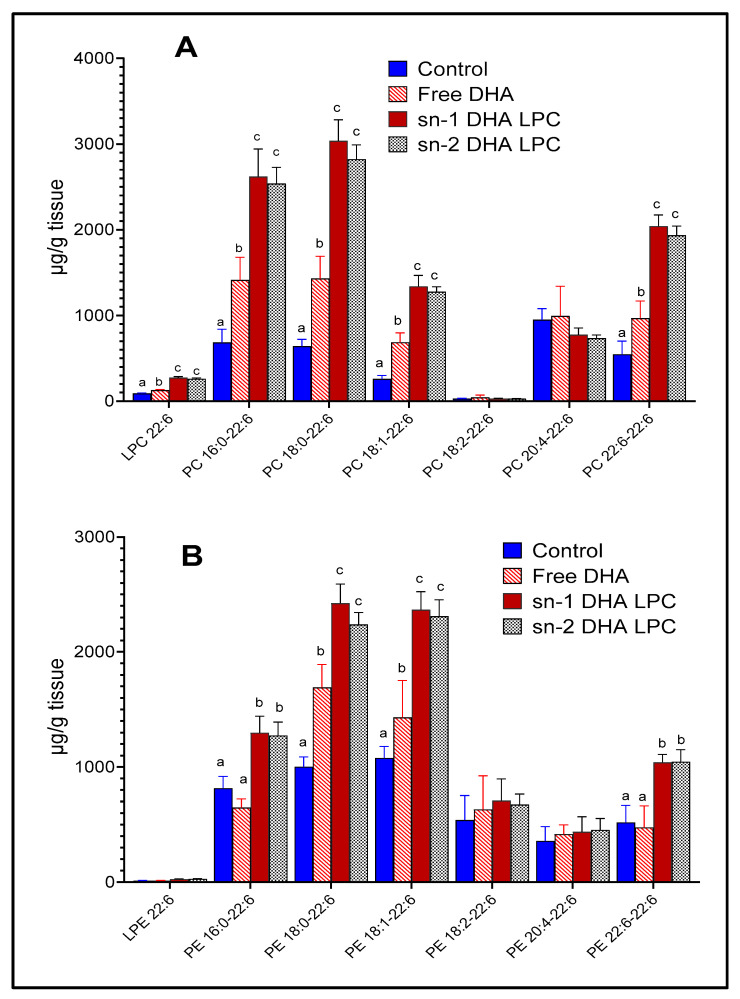
Molecular species of mouse retinal phospholipids containing DHA. The animals were gavaged with free (unesterified) DHA or two isomers of LPC-DHA as described under Figure 2. The molecular species of PC (**A**) and PE (**B**) which contain DHA were analyzed using multiple reaction monitoring by LC/MS/MS, as described in the text, using 17:0 LPC, 17:0–17:0 PC, and 17:0–17:0 PE as internal standards. Bars without common superscripts are significantly different from each other by a one-way ANOVA, and Tukey post-hoc analysis (mean ± SD), 6 animals/group). PE: phosphatidylethanolamine; PC: phosphatidylcholine; sn-1 and sn-2: stereospecific numbering 1 and 2 respectively; DHA: docosahexaenoic acid; LC/MS/MS: liquid chromatography/tandem mass spectroscopy; SD: standard deviation; LPC: lysophosphatidylcholine; DHA: docosahexaenoic acid.

The molecular species of PC and PE, which contain DHA, were analyzed by LC/MS/MS, in order to determine whether the metabolic fate of the two LPC-isomers in retina differ from each other. As shown in Figure 3, both isomers of LPC-DHA increased most of the major DHA-containing PCs and PEs, except 20:4–22:6 PC and 20:4–22:6 PE, possibly because of the decrease in retinal 20:4 by the LPC treatment. There were no significant differences between the effects of the two isomers of LPC on molecular species composition of PC or PE. Although the total DHA content of retina was not significantly increased by free DHA (Figure 2), a few individual species of PC and PE containing DHA were increased, but at much lower levels compared to LPC-DHA. Retinal LPC-DHA, but not LPE-DHA was increased after the treatment with dietary LPC-DHA. Unlike the brain, in which DHA was more prevalent in the PE species [16], retina contained more DHA in the PC species. PE: phosphatidylethanolamine.

### 3.3. Effect of Fish Oil and Krill Oil on Mouse Retinal Omega-3 FA

Although previous studies with fish oil showed no significant enrichment of retinal DHA or EPA in the adult animals [27], we recently demonstrated that pre-treatment of krill oil with a lipase, which generates LPC-EPA and LPC-DHA, enables significant enrichment of both DHA and EPA in the brains of adult mice [21]. On the other hand, similar treatment of fish oil, which generates free EPA and DHA or monoacylglycerol EPA and DHA, did not have any effect on brain omega-3 FA. In order to determine whether lipase-treated krill oil can also be used for enriching retinal DHA and EPA, we analyzed the FA composition of retina in mice treated with fish oil and krill oil, which have been treated with lipase or not.

**Figure 4:**
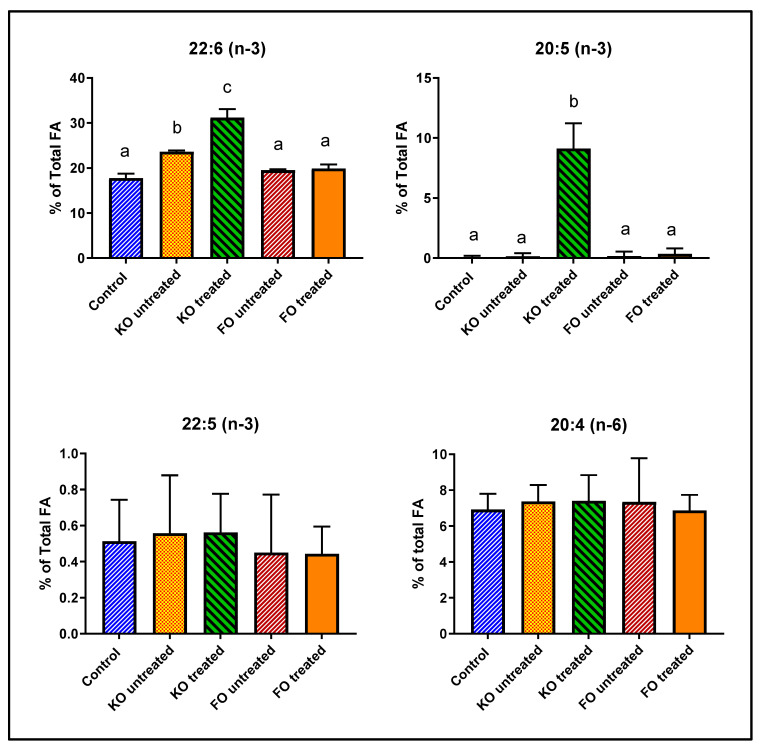
Effect of feeding untreated or lipase-treated fish oil and krill oil on mouse retinal FA. Normal male mice (8-week-old) were fed diets (AIN93G) containing 7% total fat and supplemented with 0.264% EPA+DHA in the form of fish oil or krill oil, which have been treated (or not) with *Mucor* lipase. The mice were fed the diets ad lib for 30 days and the retinal FA were analyzed by GC/MS. The percentages of 20:4 (n-6), 20:5 (n-3), 22:5 (n-3) and 22:6 (n-3) are shown here (mean ± SD, 5 mice/group. The total FA composition is shown in Table 3. Bars without common superscripts are significantly different from each other by a one-way ANOVA and Tukey post-hoc analysis. KO: krill oil; FO: fish oil; FA: fatty acid; EPA: eicosapentaenoic acid; GC/MS: gas chromatography/mass spectroscopy; DHA: docosahexaenoic acid.

As shown in Figure 4 and Table 3, retinal DHA was increased above the control value by 33% after feeding untreated krill oil, possibly because of the presence of small amounts of LPC-DHA in the krill oil preparation [21]. However, feeding lipase-treated krill oil increased the retinal DHA by 76%, showing a 2.3-fold stimulation of DHA enrichment by lipase treatment. Furthermore, there was a 100-fold increase in retinal EPA by the lipase-treated krill oil, but no increase with untreated krill oil or the fish oil. This supports our previous observation that feeding LPC-EPA does increase EPA levels in the brain and retina, contrary to the previous reports of the lack of EPA enrichment in these tissues by omega-3 FA-enriched diets [28,29,30]. In contrast to the results with pure LPC-DHA, we did not find a significant displacement of retinal arachidonate by DHA after treatment with lipase-treated krill oil. Instead, DHA and EPA appeared to replace saturated FA and oleic acid (18:1, n-9). There was also some decrease in saturated FA and 18:1 in the animals fed untreated krill oil, but these decreases did not reach statistical significance (Table 3).

## 4. Discussion

Retinal DHA is known to decline with age [3] as well as in diabetes [6,7,8]. Furthermore, the reduced DHA levels have been associated with several retinal diseases, the most prominent being diabetic retinopathy (DR) [3,5,31]. DR affects almost 100 million people world-wide and is the most common cause of blindness in the adult population [32]. It is believed that the oxidative stress and chronic inflammation induced by hyperglycemia are the major underlying causes of DR [33]. DHA, which is uniquely concentrated in retina, has been shown to have both antioxidant and anti-inflammatory properties [3,34,35]. In addition to DR, DHA deficiency has also been implicated in other diseases of the eye, including retinitis pigmentosa [36], glaucoma [37,38], age-related macular degeneration [4], dry eye disease [39], and Alzheimer’s related blindness [18]. A common element of all of these diseases is chronic inflammation. Therefore, it is important to investigate whether the retinal DHA levels can be increased through diet in adult mammals and thereby prevent or treat these diseases. The results presented here show, that despite the very high initial levels of DHA in the normal retina, it can be further increased by up to 100% through dietary LPC-DHA, but not by dietary free DHA or TAG-DHA. To our knowledge, this is the highest enrichment achieved in retinal DHA with dietary supplementation in normal adult animals. Previous studies found no or marginal increases in retinal omega-3 levels even after treatment with very high concentrations of dietary omega-3 FA. For example, Prokopiou et al. [40] fed aged (2-year-old) mice 200 mg omega-3 FA/day in the form of fish oil for 60 days, and found actually a decrease in retinal DHA (−21%), although retinal EPA, which is a minor constituent, increased by 42%. Similarly, gavaging ABCA4−/− mice (Stargardt disease) with 206 mg/day of omega-3 FA (172 mg EPA + 34 mg DHA) for three months resulted in no change in retinal DHA, although a 67% increase in retinal EPA (from 0.93% to 1.56% of total) was observed [41]. A study by Schnebelen et al. [42] showed that feeding 3-week-old rats with 5% fat diet containing 20% omega-3 FA for three months resulted only in an 8% increase in retinal DHA. In contrast to these studies, using transgenic mice carrying fat-1 gene, which converts endogenous omega-6 FA to omega-3 FA, Suh et al. [43] and Tanito et al. [44] reported a near doubling of retinal DHA compared to the wild type controls. However, the increase in retinal DHA by genetic manipulation rather than through diet appears to cause abnormal electroretinograms and susceptibility to oxidative stress, as reported by these workers. On the other hand, Connor et al. [45] reported that increasing the DHA content of retina in neonatal mice through diet prevents retinopathy of prematurity by inhibiting the pathological neovascularization. Furthermore, Sapieha et al. [5] showed that supplementation of diet with high concentration of omega-3 FA (2% of diet) preserved retinal function in a mouse model of type 2 diabetes and enhanced glucose tolerance, although changes in retinal DHA levels were not reported. Similar benefits on diabetic retinopathy were shown in rats by Tikhonenko et al. [31] who fed 5% calories as menhaden oil. However, the dose of omega-3 FA required to achieve these beneficial effects is impractical in clinical setting, since the equivalent dose in humans, using allometric calculations [46], would be about 14 g of omega-3 FA/day in a 70 kg human, based on the Sapieha et al. study, or about 16 mL fish oil/day according to the Tikhonenko et al. study. In contrast, the dose of LPC-DHA required to nearly double the retinal DHA is about 50 times lower than the above studies and is therefore easily applicable to clinical conditions.

Unlike the brain which acquires DHA predominantly through the Mfsd2a pathway, retina appears to acquire DHA through multiple pathways, since a deficiency of Mfs2a results in only a 45% reduction in retinal DHA, and a 57% reduction in VLCFA [47]. Thus, the studies by Bazan [18] showed the importance of Adiponectin receptor for maintaining the DHA levels of retina, whereas the role of FA-binding proteins and lipoprotein receptors have been proposed by others [3,19]. These pathways may account for the small increase in retinal DHA by dietary free DHA and TAG. Retina also has an efficient recycling mechanism to retain DHA by the phagocytosis of retinal pigment epithelial cells [1,18], possibly accounting for the milder effects of congenital Mfsd2a deficiency on retinal function [20,47], compared to the brain function [17]. Another difference between the brain and retina was that whereas DHA replaced mostly arachidonic acid in the brain [15,16,21], it replaced more saturated fatty acids and oleic acid than arachidonic acid in retina. While the decrease in arachidonic acid is believed to be beneficial because of its role in the generation of pro-inflammatory eicosanoids, the physiological effects of decreasing the saturated fatty acids and oleic acid in retina are not clear.

In contrast to DHA, the EPA content of retina is very low, and is not increased substantially even after feeding EPA-rich supplements, although DHA levels are increased by these treatments [42,45]. Therefore, it has been assumed that either EPA does not enter the brain and retina, convert rapidly to DHA, or oxidized without net accumulation [48,49]. In the current study, although EPA levels of retina were not increased after feeding pure LPC-DHA, marked increases occurred after feeding lipase-treated krill oil which contained both LPC-EPA and LPC-DHA. Therefore, we suggest that the failure of previous studies to show an increase in brain or retinal EPA was due to the inability of the supplements to generate LPC-EPA in vivo. We have previously shown that similar enrichment of brain EPA and retinal EPA occurred after feeding pure LPC-EPA [50]. Increasing retinal EPA in addition to DHA may be more beneficial than increasing only its DHA content, because EPA is the preferred substrate for the synthesis of VLCFA [51,52], which have unique functional significance in retina [53]. EPA is also known to compete more effectively than DHA against arachidonate and thereby inhibit synthesis of pro-inflammatory prostaglandins.

Most studies on omega-3 FA focus on increasing only the EPA and DHA levels of the tissues, but an ancillary benefit of dietary LPC-EPA/DHA, in comparison to fish oil or ethyl esters, is that for each molecule of DHA or EPA taken up by the retina and brain through the Mfsd2a pathway, a molecule of choline is simultaneously taken up. Choline is an essential component of acetyl choline as well as membrane phospholipids, and plays a critical role in vision [54]. In fact, citicholine, (CDP-choline), a precursor of choline phospholipids, is used clinically for treatment of retinopathies and glaucoma [54]. Therefore, LPC-EPA/DHA could provide the combined benefits of omega-3 FA and citicholine in a single effective preparation.

## Figures and Tables

**Table 1:** Effect of dietary TG-DHA, di-DHA PC, and LPC-DHA on rat retinal fatty acid composition.

	Control			TG-DHA				PC-DHA				LPC-DHA 5 mg				LPC-DHA 10 mg			
F.A.	Mean		S.D.	Mean		S.D.		Mean		S.D.		Mean		S.D.		Mean		S.D.	
12:0	4.39	±	1.19	4.71	±	2.09		4.57	±	1.49		3.10	±	0.08		3.10	±	0.60	
14:0	0.08	±	0.04	0.17	±	0.18		0.97	±	1.97		0.52	±	0.90		0.11	±	0.13	
16:0	22.14	±	0.65	21.52	±	1.37		19.06	±	0.76	**	18.05	±	0.84	**	17.27	±	0.94	**
16:1 (n-7)	0.73	±	0.06	0.74	±	0.09		0.65	±	0.04		0.66	±	0.08		0.68	±	0.05	
17:1 (n-7)	0.02	±	0.02	0.12	±	0.03	*	0.03	±	0.02		0.01	±	0.00		0.02	±	0.02	
18:0	20.26	±	0.92	19.09	±	1.30		17.79	±	0.97		16.84	±	0.43	**	15.62	±	0.85	**
18:1 (n-9)	18.98	±	0.90	18.21	±	1.57		17.70	±	1.26		17.07	±	0.94		15.81	±	1.46	
18:1(n-7)	4.40	±	0.28	4.09	±	0.16		3.91	±	0.25		3.79	±	0.16		3.43	±	0.28	*
18:2 (n-6)	0.97	±	0.05	1.05	±	0.36		0.95	±	0.22		0.93	±	0.26		0.97	±	0.26	
18:3 (n-6)	0.39	±	0.02	0.38	±	0.11		0.49	±	0.13		0.50	±	0.11		0.37	±	0.08	
18:3 (n-3)	0.03	±	0.02	0.09	±	0.10		0.07	±	0.07		0.08	±	0.05		0.04	±	0.04	
20:0	0.05	±	0.04	0.08	±	0.05		0.05	±	0.03		0.04	±	0.03		0.04	±	0.03	
20:1 (n-9)	0.05	±	0.05	0.06	±	0.05		0.07	±	0.03		0.05	±	0.03		0.03	±	0.02	
20:2 (n-6)	0.08	±	0.04	0.04	±	0.01		0.06	±	0.05		0.05	±	0.04		0.06	±	0.04	
20:3 (n-6)	0.08	±	0.10	0.16	±	0.18		0.06	±	0.04		0.09	±	0.13		0.13	±	0.15	
20:4 (n-6)	9.77	±	0.44	9.49	±	0.52		8.45	±	0.67		7.94	±	0.48	*	7.44	±	0.37	**
22:0	0.04	±	0.02	0.05	±	0.04		0.07	±	0.07		0.03	±	0.02		0.04	±	0.02	
20:5 (n-3)	0.07	±	0.09	0.08	±	0.04		0.06	±	0.05		0.04	±	0.03		0.05	±	0.04	
22:2 (n-6)	0.11	±	0.08	0.13	±	0.06		0.12	±	0.05		0.12	±	0.04		0.13	±	0.03	
22:4 (n-6)	0.05	±	0.03	0.13	±	0.17		0.07	±	0.05		0.24	±	0.45		0.32	±	0.62	
22:5 (n-6)	0.02	±	0.01	0.03	±	0.04		0.02	±	0.01		0.02	±	0.03		0.03	±	0.02	
22:5 (n-3)	0.13	±	0.11	0.09	±	0.09		0.06	±	0.04		0.06	±	0.06		0.14	±	0.12	
22:6 (n-3)	16.88	±	1.15	19.20	±	1.67		24.53	±	2.73	*	29.56	±	1.23	**	33.98	±	1.91	**
24:1 (n-9)	0.06	±	0.04	0.07	±	0.04		0.05	±	0.02		0.03	±	0.02		0.03	±	0.02	
16:0 DMA	0.03	±	0.03	0.09	±	0.08		0.05	±	0.08		0.12	±	0.12		0.08	±	0.10	
18:0 DMA	0.16	±	0.30	0.05	±	0.03		0.03	±	0.02		0.03	±	0.02		0.02	±	0.01	
18:1 DMA	0.06	±	0.05	0.08	±	0.08		0.06	±	0.04		0.05	±	0.02		0.06	±	0.06	

* *p* < 0.05 compared to control, ** *p* < 0.005 compared to control, unpaired *t*-test adjusted with Holm–Sidak method. TG: triacylglycerol; PC: phosphatidylcholine; LPC: lysophosphatidylcholine; DMA: dimethylacetal; DHA: docosahexaenoic acid; F.A.: fatty acid; S.D.: standard deviation.

**Table 2:** Effect of free DHA, sn-1 DHA LPC, and sn-2 DHA LPC on fatty acid composition of mouse retina.

	Control			Free DHA			sn-1 DHA LPC				sn-2 DHA LPC			
F.A.	Mean	±	S.D.	Mean	±	S.D.	Mean	±	S.D.		Mean	±	S.D.	
12:0	0.26	±	0.14	0.60	±	0.35	0.73	±	0.47		0.73	±	0.47	
14:0	0.50	±	0.25	0.20	±	0.17	0.30	±	0.17		0.30	±	0.17	
16:0	17.92	±	1.41	16.11	±	1.56	14.62	±	1.22	**	14.62	±	1.22	**
16:1 (n-7)	0.38	±	0.17	0.55	±	0.28	0.47	±	0.34		0.47	±	0.34	
17:1 (n-7)	0.29	±	0.13	0.43	±	0.16	0.48	±	0.38		0.48	±	0.38	
18:0	18.90	±	1.05	17.32	±	0.66	15.44	±	1.50	**	15.44	±	1.50	**
18:1 (n-9)	18.23	±	0.82	17.14	±	0.78	15.56	±	1.16	**	15.56	±	1.16	**
18:1(n-7)	4.64	±	0.48	4.18	±	0.34	3.82	±	0.62		3.82	±	0.62	
18:2 (n-6)	0.69	±	0.45	0.99	±	0.65	1.38	±	0.49		1.38	±	0.49	
18:3 (n-6)	0.36	±	0.19	0.49	±	0.36	0.60	±	0.47		0.60	±	0.47	
18:3 (n-3)	0.54	±	0.43	0.49	±	0.15	0.51	±	0.42		0.51	±	0.42	
20:0	1.86	±	0.69	1.90	±	0.64	1.18	±	0.72		1.18	±	0.72	
20:1 (n-9)	3.71	±	0.42	3.42	±	0.59	2.87	±	0.60		2.87	±	0.60	
20:2 (n-6)	1.28	±	0.84	0.71	±	0.50	0.88	±	0.95		0.88	±	0.95	
20:3 (n-6)	0.69	±	0.54	1.30	±	0.66	1.44	±	0.35		1.44	±	0.35	
20:4 (n-6)	7.86	±	0.81	7.42	±	0.72	5.75	±	0.71	**	5.75	±	0.71	**
22:0	1.20	±	0.41	1.08	±	0.59	1.24	±	0.95		1.24	±	0.95	
20:5 (n-3)	0.86	±	0.53	2.03	±	1.21	1.91	±	1.40		1.91	±	1.40	
22:2 (n-6)	0.80	±	0.34	0.77	±	0.51	1.00	±	0.76		1.00	±	0.76	
22:4 (n-6)	2.03	±	0.91	2.51	±	0.85	2.23	±	0.87		2.23	±	0.87	
22:5 (n-3)	0.48	±	0.44	0.42	±	0.15	0.79	±	0.76		0.79	±	0.76	
22:6 (n-3)	12.61	±	0.91	15.74	±	1.57	22.57	±	3.12	**	22.57	±	3.12	**
24:1 (n-9)	0.78	±	0.48	0.51	±	0.25	0.49	±	0.17		0.49	±	0.17	
16:0 DMA	1.16	±	0.96	0.75	±	0.56	1.02	±	0.32		1.02	±	0.32	
18:0 DMA	1.25	±	1.04	2.10	±	0.88	1.96	±	0.30		1.96	±	0.30	
18:1 DMA	0.73	±	0.75	0.85	±	0.56	0.78	±	0.64		0.78	±	0.64	

** *p* < 0.005 compared to control, unpaired *t*-test adjusted with Holm–Sidak method. DMA: dimethylacetal; sn-1 and sn-2: stereospecific numbering 1 and 2 respectively; LPC: lysophosphatidylcholine; DHA: docosahexaenoic acid; F.A.: fatty acid; S.D.: standard deviation.

**Table 3:** Effect of feeding unmodified, and lipase-treated krill oil and fish oil on mouse retina fatty acid composition.

	Control			KO Untreated			KO Treated				FO Untreated			FO Treated		
F.A.	Mean		S.D.	Mean		S.D.	Mean		S.D.		Mean		S.D.	Mean		S.D
12:0	0.02	±	0.03	0.06	±	0.06	0.07	±	0.05		0.02	±	0.01	0.06	±	0.05
14:0	0.17	±	0.27	0.38	±	0.44	0.09	±	0.04		0.20	±	0.33	0.03	±	0.04
16:0	22.07	±	1.84	20.34	±	1.94	12.51	±	7.01		22.20	±	1.90	22.67	±	2.12
16:1 (n-7)	0.20	±	0.31	0.19	±	0.17	0.10	±	0.13		0.06	±	0.07	0.04	±	0.04
18:0	18.69	±	0.42	17.20	±	1.25	14.20	±	1.62	*	17.84	±	0.88	18.03	±	0.13
18:1 (n-9)	20.04	±	0.64	14.99	±	5.59	14.75	±	1.50	**	19.51	±	0.76	19.32	±	0.99
18:1(n-7)	4.79	±	0.21	4.36	±	0.42	2.35	±	2.08		4.46	±	0.14	3.72	±	2.09
18:2 (n-6)	0.88	±	0.46	0.99	±	0.51	1.10	±	0.34		1.22	±	0.19	0.87	±	0.69
18:3 (n-6)	0.06	±	0.04	0.06	±	0.06	0.18	±	0.16		0.06	±	0.05	0.05	±	0.01
18:3 (n-3)	0.81	±	0.53	0.82	±	0.74	0.29	±	0.24		0.79	±	0.68	0.41	±	0.31
20:0	0.05	±	0.03	0.03	±	0.03	0.04	±	0.04		0.05	±	0.03	0.02	±	0.01
20:1 (n-9)	2.17	±	1.17	2.00	±	1.41	0.78	±	0.44		2.13	±	0.55	1.12	±	0.92
20:2 (n-6)	0.22	±	0.19	0.38	±	0.25	0.19	±	0.15		0.56	±	0.39	0.21	±	0.10
20:3 (n-6)	0.03	±	0.02	0.11	±	0.13	0.44	±	0.41		0.05	±	0.02	0.23	±	0.41
20:4 (n-6)	6.92	±	0.88	7.37	±	0.92	7.41	±	1.43		7.34	±	2.44	6.87	±	0.86
22:0	0.10	±	0.10	0.37	±	0.32	0.09	±	0.10		0.14	±	0.25	0.06	±	0.06
20:5 (n-3)	0.09	±	0.10	0.15	±	0.26	9.12	±	2.09	**	0.18	±	0.36	0.36	±	0.45
22:2 (n-6)	0.09	±	0.09	0.20	±	0.18	0.15	±	0.16		0.12	±	0.13	0.14	±	0.15
22:4 (n-6)	1.11	±	1.40	2.56	±	1.44	1.79	±	1.55		0.55	±	1.10	2.76	±	0.53
22:5 (n-6)	0.06	±	0.04	0.15	±	0.09	0.24	±	0.20		0.17	±	0.19	0.06	±	0.03
22:5 (n-3)	0.51	±	0.23	0.56	±	0.32	0.56	±	0.22		0.45	±	0.32	0.44	±	0.15
22:6 (n-3)	17.76	±	1.00	23.62	±	0.26	31.18	±	1.89	**	19.52	±	0.18	19.89	±	0.91
24:1 (n-9)	0.04	±	0.03	0.11	±	0.13	0.09	±	0.06		0.04	±	0.01	0.03	±	0.03
16:0 DMA	0.05	±	0.02	0.05	±	0.03	0.06	±	0.04		0.13	±	0.17	0.03	±	0.02
18:0 DMA	2.96	±	0.467	2.642	±	0.95	2.126	±	0.56		1.97	±	0.67	2.48	±	0.47
18:1 DMA	0.02	±	0.02	0.05	±	0.05	0.04	±	0.04		0.02	±	0.02	0.03	±	0.03

* *p* < 0.05 compared to control, ** *p* < 0.005 compared to control, unpaired *t*-test adjusted with Holm–Sidak method. KO: krill oil; FO: fish oil; FA: fatty acid; S.D.: standard deviation.

## References

[1] Stinson A.M., Wiegand R.D., Anderson R.E. (1991). Recycling of docosahexaenoic acid in rat retinas during n-3 fatty acid deficiency. J. Lipid Res..

[2] Jasani B., Simmer K., Patole S.K., Rao S.C. (2017). Long chain polyunsaturated fatty acid supplementation in infants born at term. Cochr. Database Syst. Rev..

[3] SanGiovanni J.P., Chew E.Y. (2005). The role of omega-3 long-chain polyunsaturated fatty acids in health and disease of the retina. Progress Retin. Eye Res..

[4] Souied E.H., Aslam T., Garcia-Layana A., Holz F.G., Leys A., Silva R., Delcourt C. (2016). Omega-3 fatty acids and age-related macular degeneration. Ophthalmic Res..

[5] Sapieha P., Chen J., Stahl A., Seaward M.R., Favazza T.L., Juan A.M., Hatton C.J., Joyal J.S., Krah N.M., Dennison R.J. (2012). Omega-3 polyunsaturated fatty acids preserve retinal function in type 2 diabetic mice. Nutr. Diabet..

[6] Yee P., Weymouth A.E., Fletcher E.L., Vingrys A.J. (2010). A Role for Omega-3 Polyunsaturated Fatty Acid Supplements in Diabetic Neuropathy. Investig. Ophthalmol. Vis. Sci..

[7] Hegde K.R., Varma S.D. (2009). Electron impact mass spectroscopic studies on mouse retinal fatty acids: Effect of diabetes. Ophthalmic Res..

[8] Futterman S., Sturtevant R., Kupfer C. (1969). Effect of alloxan diabetes on the fatty acid composition of the retina. Investig. Ophtalmol. Vis. Sci..

[9] Anderson R.E., Maude M.B., Bok D. (2001). Low docosahexaenoic acid levels in rod outer segment membranes of mice with rds/peripherin and P216L peripherin mutations. Investig. Ophthalmol. Vis. Sci..

[10] Gong J., Rosner B., Rees D.G., Berson E.L., Weigel-DiFranco C.A., Schaefer E.J. (1992). Plasma docosahexaenoic acid levels in various genetic forms of retinitis pigmentosa. Investig. Ophthalmol. Vis. Sci..

[11] Martínez M. (1990). Severe deficiency of docosahexaenoic acid in peroxisomal disorders: A defect of delta 4 desaturation?. Neurology.

[12] Uauy R., Hoffman D.R., Peirano P., Birch D.G., Birch E.E. (2001). Essential fatty acids in visual and brain development. Lipids.

[13] Hoffman D.R., Hughbanks-Wheaton D.K., Pearson N.S., Fish G.E., Spencer R., Takacs A., Klein M., Locke K.G., Birch D.G. (2014). Four-year placebo-controlled trial of docosahexaenoic acid in X-linked retinitis pigmentosa (DHAX trial): A randomized clinical trial. JAMA Ophthalmol..

[14] Souied E.H., Delcourt C., Querques G., Bassols A., Merle B., Zourdani A., Smith T., Benlian P. (2013). Oral docosahexaenoic acid in the prevention of exudative age-related macular degeneration: The nutritional AMD treatment 2 study. Ophthalmology.

[15] Sugasini D., Yalagala P.C.R., Goggin A., Tai L.M., Subbaiah P.V. (2019). Enrichment of brain docosahexaenoic acid (DHA) is highly dependent upon the molecular carrier of dietary DHA: Lysophosphatidylcholine is more efficient than either phosphatidylcholine or triacylglycerol. J. Nutr. Biochem..

[16] Sugasini D., Thomas R., Yalagala P.C.R., Tai L.M., Subbaiah P.V. (2017). Dietary docosahexaenoic acid (DHA) as lysophosphatidylcholine, but not as free acid, enriches brain DHA and improves memory in adult mice. Sci. Rep..

[17] Nguyen L.N., Ma D., Shui G., Wong P., Cazenave-Gassiot A., Zhang X., Wenk M.R., Goh E.L.K., Silver D.L. (2014). Mfsd2a is a transporter for the essential omega-3 fatty acid docosahexaenoic acid. Nature.

[18] Bazan N.G. (2018). Docosanoids and elovanoids from omega-3 fatty acids are pro-homeostatic modulators of inflammatory responses, cell damage and neuroprotection. Mol. Asp. Med..

[19] Tachikawa M., Akanuma S.I., Imai T., Okayasu S., Tomohiro T., Hatanaka Y., Hosoya K.I. (2018). Multiple cellular transport and binding processes of unesterified docosahexaenoic acid in outer blood-retinal barrier retinal pigment epithelial cells. Biol. Pharm. Bull..

[20] Wong B.H., Chan J.P., Cazenave-Gassiot A., Poh R.W., Foo J.C., Galam D.L., Ghosh S., Nguyen L.N., Barathi V.A., Yeo S.W. (2016). Mfsd2a is a transporter for the essential omega-3 fatty acid docosahexaenoic acid (DHA) in eye and is important for photoreceptor cell development. J. Biol. Chem..

[21] Yalagala P.C.R., Sugasini D., Zaldua S.B., Tai L.M., Subbaiah P.V. (2020). Lipase treatment of dietary krill oil, but not fish oil, enables enrichment of brain eicosapentaenoic acid (EPA) and docosahexaenoic acid (DHA). Mol. Nutr. Food Res..

[22] Bligh E.G., Dyer W.J. (1959). A rapid method of total lipid extraction and purification. Can. J. Biochem. Physiol..

[23] Ivanova P.T., Milne S.B., Byrne M.O., Xiang Y., Brown H.A. (2007). Glycerophospholipid identification and quantitation by electrospray ionization mass spectrometry. Methods Enzymol..

[24] Subbaiah P.V., Dammanahalli K.J., Yang P., Bi J., O’Donnell J.M. (2016). Enhanced incorporation of dietary DHA into lymph phospholipids by altering its molecular carrier. Biochim. Biophys. Acta.

[25] Hachem M., Geloen A., Van A., Foumaux B., Fenart L., Gosselet F., Da Silva P., Breton G., Lagarde M., Picq M. (2015). Efficient docosahexaenoic acid uptake by the brain from a structured phospholipid. Mol. Neurobiol..

[26] Thies F., Delachambre M.C., Bentejac M., Lagarde M., Lecerf J. Lyso-sn 1 Phosphatidylcholine Bound to Albumin: A Preferential Form for Rat Brain Uptake of Unsaturated Fatty Acids Compared to the Unesterified Form?. Proceedings of the 32nd International Conference on Biochemistry of Lipids.

[27] Nishizawa C., Wang J.-Y., Sekine S., Saito M. (2003). Effect of dietary DHA on DHA levels in retinal rod outer segments in young versus mature rats. Int. J. Vitam. Nutr. Res..

[28] Rodrigues P.O., Martins S.V., Lopes P.A., Miguueis S., Alfaia C.M., Pinto R.M.A., Rolo E.A., Bispo P., Batista I., Bandarra N.M. (2014). Influence of feeding graded levels of canned sardines on the inflammatory markers and tissue fatty acid composition of Wistar rats. Br. J. Nutr..

[29] Kaur G., Begg D.P., Barr D., Garg M., Cameron-Smith D., Sinclair A.J. (2010). Short-term docosapentaenoic acid (22:5 n-3) supplementation increases tissue docosapentaenoic acid, DHA and EPA concentrations in rats. Br. J. Nutr..

[30] Tou J., Altman S., Gigliotti J., Benedito V., Cordonier E. (2011). Different sources of omega-3 polyunsaturated fatty acids affects apparent digestibility, tissue deposition, and tissue oxidative stability in growing female rats. Lipids Health Dis..

[31] Tikhonenko M., Lydic T.A., Opreanu M., Li Calzi S., Bozack S., McSorley K.M., Sochacki A.L., Faber M.S., Hazra S., Duclos S. (2013). N-3 Polyunsaturated fatty acids prevent diabetic retinopathy by inhibition of retinal vascular damage and enhanced endothelial progenitor cell reparative function. PLoS ONE.

[32] Abcouwer S.F., Gardner T.W. (2014). Diabetic retinopathy: Loss of neuroretinal adaptation to the diabetic metabolic environment. Ann. N. Y. Acad. Sci..

[33] Rossino M.G., Casini G. (2019). Nutraceuticals for the treatment of diabetic retinopathy. Nutrients.

[34] Hashimoto M., Hossain S., Al Mamun A., Matsuzaki K., Arai H. (2017). Docosahexaenoic acid: One molecule diverse functions. Crit. Rev. Biotechnol..

[35] Green P., Glozman S., Weiner L., Yavin E. (2001). Enhanced free radical scavenging and decreased lipid peroxidation in the rat fetal brain after treatment with ethyl docosahexaenoate. Biochim. Biophys. Acta Mol. Cell Biol. Lipids.

[36] Schaefer E.J., Robins S.J., Patton G.M., Sandberg M.A., Weigel-DiFranco C.A., Rosner B., Berson E.L. (1995). Red blood cell membrane phosphatidylethanolamine fatty acid content in various forms of retinitis pigmentosa. J. Lipid Res..

[37] Kalogerou M., Kolovos P., Prokopiou E., Papagregoriou G., Deltas C., Malas S., Georgiou T. (2018). Omega-3 fatty acids protect retinal neurons in the DBA/2J hereditary glaucoma mouse model. Exp. Eye Res..

[38] Yang S.P., Morita I., Murota S.I. (1998). Eicosapentaenoic acid attenuates vascular endothelial growth factor-induced proliferation via inhibiting Flk-1 receptor expression in bovine carotid artery endothelial cells. J. Cell Physiol..

[39] McCusker M.M., Durrani K., Payette M.J., Suchecki J. (2016). An eye on nutrition: The role of vitamins, essential fatty acids, and antioxidants in age-related macular degeneration, dry eye syndrome, and cataract. Clin. Dermatol..

[40] Prokopiou E., Kolovos P., Georgiou C., Kalogerou M., Potamiti L., Sokratous K., Kyriacou K., Georgiou T. (2019). Omega-3 fatty acids supplementation protects the retina from age-associated degeneration in aged C57BL/6J mice. BMJ Open Ophthalmol..

[41] Prokopiou E., Kolovos P., Kalogerou M., Neokleous A., Nicolaou O., Sokratous K., Kyriacou K., Georgiou T. (2018). Omega-3 fatty acids supplementation: Therapeutic potential in a mouse model of stargardt disease. Investig. Ophtalmol. Vis. Sci..

[42] Schnebelen C., Viau S., Grégoire S., Joffre C., Creuzot-Garcher C.P., Bron A.M., Bretillon L., Acar N. (2009). Nutrition for the eye: Different susceptibility of the retina and the lacrimal gland to dietary omega-6 and omega-3 polyunsaturated fatty acid incorporation. Ophthalmic Res..

[43] Tanito M., Brush R.S., Elliott M.H., Wicker L.D., Henry K.R., Anderson R.E. (2009). High levels of retinal membrane docosahexaenoic acid increase susceptibility to stress-induced degeneration. J. Lipid Res..

[44] Suh M., Sauvé Y., Merrells K.J., Kang J.X., Ma D.W.L. (2009). Supranormal electroretinogram in F at-1 mice with retinas enriched in docosahexaenoic acid and n-3 very long chain fatty acids (C24–C36). Investig. Ophtalmol. Vis. Sci..

[45] Connor K.M., SanGiovanni J.P., Lofqvist C., Aderman C.M., Chen J., Higuchi A., Hong S., Pravda E.A., Majchrzak S., Carper D. (2007). Increased dietary intake of -ë-3-polyunsaturated fatty acids reduces pathological retinal angiogenesis. Nat. Med..

[46] Nair A.B., Jacob S. (2016). A simple practice guide for dose conversion between animals and human. J. Basic Clin. Pharm..

[47] Lobanova E.S., Schuhmann K., Finkelstein S., Lewis T.R., Cady M.A., Hao Y., Keuthan C., Ash J.D., Burns M.E., Shevchenko A. (2019). Disrupted blood-retina lysophosphatidylcholine transport impairs photoreceptor health but not visual signal transduction. J. Neurosci..

[48] Chen C.T., Bazinet R.P. (2015). b-oxidation and rapid metabolism, but not uptake regulate brain eicosapentaenoic acid levels. Prostaglandins Leukot. Essent. Fatty Acids.

[49] Kaur G., Molero J.C., Weisinger H.S., Sinclair A.J. (2013). Orally administered [14C] DPA and [14C] DHA are metabolised differently to [14C] EPA in rats. Br. J. Nutr..

[50] Yalagala P.C.R., Sugasini D., Dasarathi S., Pahan K., Subbaiah P.V. (2019). Dietary lysophosphatidylcholine-EPA enriches both EPA and DHA in the brain: Potential treatment for depression. J. Lipid Res..

[51] Suh M., Clandinin M.T. (2005). 20:5n-3 but not 22:6n-3 is a preferred substrate for synthesis of n-3 very-long-chain fatty acids (C24–C36) in retina. Curr. Eye Res..

[52] Yu M., Benham A., Logan S., Brush R.S., Mandal M.N., Anderson R.E., Agbaga M.P. (2012). ELOVL4 protein preferentially elongates 20:5n3 to very long chain PUFAs over 20:4n6 and 22:6n3. J. Lipid Res..

[53] Jun B., Mukherjee P.K., Asatryan A., Kautzmann M.A., Heap J., Gordon W.C., Bhattacharjee S., Yang R., Petasis N.A., Bazan N.G. (2017). Elovanoids are novel cell-specific lipid mediators necessary for neuroprotective signaling for photoreceptor cell integrity. Sci. Rep..

[54] Faiq M.A., Wollstein G., Schuman J.S., Chan K.C. (2019). Cholinergic nervous system and glaucoma: From basic science to clinical applications. Prog. Retin. Eye Res..

