# Correction: Sugasini et al. Efficient Enrichment of Retinal DHA with Dietary Lysophosphatidylcholine-DHA: Potential Application for Retinopathies. *Nutrients* 2020, *12*, 3114

**DOI:** 10.3390/nu13072166

**Published:** 2021-06-24

**Authors:** Dhavamani Sugasini, Poorna C. R. Yalagala, Papasani V. Subbaiah

**Affiliations:** 1 Department of Medicine, Section of Endocrinology and Metabolism, University of Illinois at Chicago, Chicago, IL 60612, USA; sugasini@uic.edu (D.S.); Yalagala@uic.edu (P.C.R.Y.); 2 Jesse Brown VA Medical Center, Chicago, IL 60612, USA

The authors wish to make the following corrections to their recently published paper [1].

Error in Figure 2

In Figure 2 of the original article, the data for sn-1DHA LPC were repeated by mistake under sn-2 DHA LPC.

Original Figure 2.


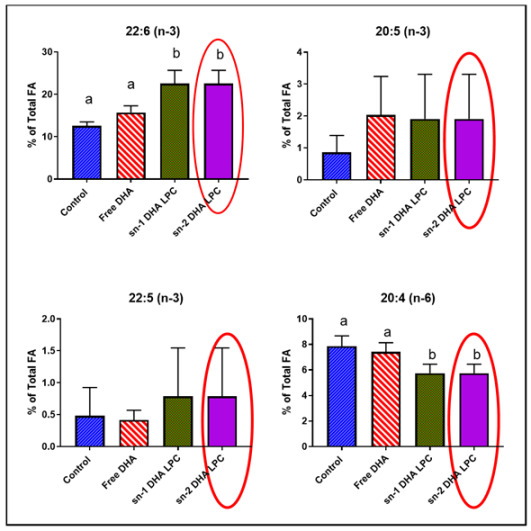


The corrected Figure 2 should be as shown below.


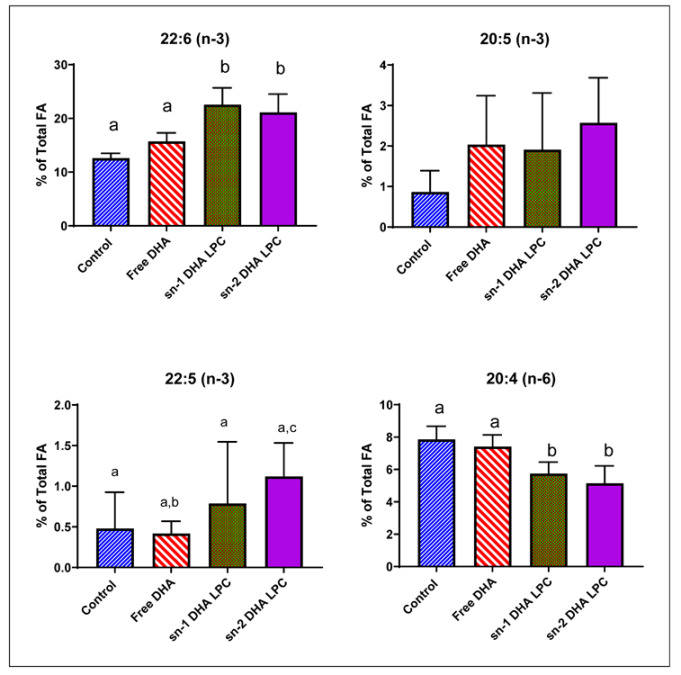


Error in Table 2

In Table 2 of the original article, the data for sn-1 DHA LPC was repeated by mistake under sn-2 DHA LPC. 

Original Table 2.

The corrected Table 2 should appear as below.

These changes have no material impact on the conclusions of the paper. The authors would like to apologize to readers of Nutrients for this error. The published version will be updated on the article webpage, with a reference to this correction notice.

## Figures and Tables

**Table 2:** Effect of free DHA, sn-1 DHA LPC, and sn-2 DHA LPC on fatty acid composition of mouse retina.

	Control			Free DHA			sn-1 DHA LPC				sn-2 DHA LPC			
FA	Mean	±	SD	Mean	±	SD	Mean	±	SD		Mean	±	SD	
12:0	0.26	±	0.14	0.60	±	0.35	0.73	±	0.47		0.73	±	0.47	
14:0	0.50	±	0.25	0.20	±	0.17	0.30	±	0.17		0.30	±	0.17	
16:0	17.92	±	1.41	16.11	±	1.56	14.62	±	1.22	**	14.62	±	1.22	**
16:1	0.38	±	0.17	0.55	±	0.28	0.47	±	0.34		0.47	±	0.34	
17:1	0.29	±	0.13	0.43	±	0.16	0.48	±	0.38		0.48	±	0.38	
18:0	18.90	±	1.05	17.32	±	0.66	15.44	±	1.50	**	15.44	±	1.50	**
18:1 (n-9)	18.23	±	0.82	17.14	±	0.78	15.56	±	1.16	**	15.56	±	1.16	**
18:1(n-7)	4.64	±	0.48	4.18	±	0.34	3.82	±	0.62		3.82	±	0.62	
18:2 (n-6)	0.69	±	0.45	0.99	±	0.65	1.38	±	0.49		1.38	±	0.49	
18:3 (n-6)	0.36	±	0.19	0.49	±	0.36	0.60	±	0.47		0.60	±	0.47	
18:3 (n-3)	0.54	±	0.43	0.49	±	0.15	0.51	±	0.42		0.51	±	0.42	
20:0	1.86	±	0.69	1.90	±	0.64	1.18	±	0.72		1.18	±	0.72	
20:1 (n-9)	3.71	±	0.42	3.42	±	0.59	2.87	±	0.60		2.87	±	0.60	
20:2 (n-6)	1.28	±	0.84	0.71	±	0.50	0.88	±	0.95		0.88	±	0.95	
20:3 (n-6)	0.69	±	0.54	1.30	±	0.66	1.44	±	0.35		1.44	±	0.35	
20:4 (n-6)	7.86	±	0.81	7.42	±	0.72	5.75	±	0.71	**	5.75	±	0.71	**
22:0	1.20	±	0.41	1.08	±	0.59	1.24	±	0.95		1.24	±	0.95	
20:5 (n-3)	0.86	±	0.53	2.03	±	1.21	1.91	±	1.40		1.91	±	1.40	
22:2	0.80	±	0.34	0.77	±	0.51	1.00	±	0.76		1.00	±	0.76	
22:4 (n-6)	2.03	±	0.91	2.51	±	0.85	2.23	±	0.87		2.23	±	0.87	
22:5 (n-3)	0.48	±	0.44	0.42	±	0.15	0.79	±	0.76		0.79	±	0.76	
22:6 (n-3)	12.61	±	0.91	15.74	±	1.57	22.57	±	3.12	**	22.57	±	3.12	**
24:1	0.78	±	0.48	0.51	±	0.25	0.49	±	0.17		0.49	±	0.17	
16:0 DMA	1.16	±	0.96	0.75	±	0.56	1.02	±	0.32		1.02	±	0.32	
18:0 DMA	1.25	±	1.04	2.10	±	0.88	1.96	±	0.30		1.96	±	0.30	
18:1 DMA	0.73	±	0.75	0.85	±	0.56	0.78	±	0.64		0.78	±	0.64	

** *p* < 0.005 compared to control, unpaired *t*-test adjusted with Holm–Sidak method. DMA: dimethylacetal; sn-1 and sn-2: stereospecific numbering 1 and 2 respectively; LPC: lysophosphatidylcholine; DHA: docosahexaenoic acid; F.A.: fatty acid; S.D.: standard deviation.

**Table 2:** Effect of free DHA, sn-1 DHA LPC, and sn-2 DHA LPC on fatty acid composition of mouse retina.

	Control			Free DHA			sn-1 DHA LPC				sn-2 DHA LPC			
FA	Mean	±	SD	Mean	±	SD	Mean	±	SD		Mean	±	SD	
12:0	0.26	±	0.14	0.60	±	0.35	0.73	±	0.47		0.98	±	0.34	
14:0	0.50	±	0.25	0.20	±	0.17	0.30	±	0.17		0.33	±	0.25	
16:0	17.92	±	1.41	16.11	±	1.56	14.62	±	1.22	**	15.05	±	1.56	**
16:1	0.38	±	0.17	0.55	±	0.28	0.47	±	0.34		0.45	±	0.37	
17:1	0.29	±	0.13	0.43	±	0.16	0.48	±	0.38		0.61	±	0.47	
18:0	18.90	±	1.05	17.32	±	0.66	15.44	±	1.50	**	15.40	±	0.58	**
18:1 (n-9)	18.23	±	0.82	17.14	±	0.78	15.56	±	1.16	**	15.69	±	1.10	**
18:1(n-7)	4.64	±	0.48	4.18	±	0.34	3.82	±	0.62		3.87	±	0.61	
18:2 (n-6)	0.69	±	0.45	0.99	±	0.65	1.38	±	0.49		1.05	±	0.40	
18:3 (n-6)	0.36	±	0.19	0.49	±	0.36	0.60	±	0.47		0.28	±	0.13	
18:3 (n-3)	0.54	±	0.43	0.49	±	0.15	0.51	±	0.42		0.49	±	0.37	
20:0	1.86	±	0.69	1.90	±	0.64	1.18	±	0.72		2.13	±	1.71	
20:1 (n-9)	3.71	±	0.42	3.42	±	0.59	2.87	±	0.60		2.62	±	0.48	
20:2 (n-6)	1.28	±	0.84	0.71	±	0.50	0.88	±	0.95		1.06	±	0.76	
20:3 (n-6)	0.69	±	0.54	1.30	±	0.66	1.44	±	0.35		1.16	±	0.77	
20:4 (n-6)	7.86	±	0.81	7.42	±	0.72	5.75	±	0.71	**	5.14	±	1.08	**
22:0	1.20	±	0.41	1.08	±	0.59	1.24	±	0.95		1.27	±	1.14	
20:5 (n-3)	0.86	±	0.53	2.03	±	1.21	1.91	±	1.40		2.57	±	1.11	
22:2	0.80	±	0.34	0.77	±	0.51	1.00	±	0.76		1.32	±	0.80	
22:4 (n-6)	2.03	±	0.91	2.51	±	0.85	2.23	±	0.87		2.10	±	0.65	
22:5 (n-3)	0.48	±	0.44	0.42	±	0.15	0.79	±	0.76		1.12	±	0.41	
22:6 (n-3)	12.61	±	0.91	15.74	±	1.57	22.57	±	3.12	**	21.14	±	3.39	**
24:1	0.78	±	0.48	0.51	±	0.25	0.49	±	0.17		0.54	±	0.40	
16:0 DMA	1.16	±	0.96	0.75	±	0.56	1.02	±	0.32		0.98	±	0.47	
18:0 DMA	1.25	±	1.04	2.10	±	0.88	1.96	±	0.30		1.78	±	1.04	
18:1 DMA	0.73	±	0.75	0.85	±	0.56	0.78	±	0.64		0.89	±	0.51	

** *p* < 0.005 compared to control, unpaired *t*-test adjusted with Holm–Sidak method. DMA: dimethylacetal; sn-1 and sn-2: stereospecific numbering 1 and 2 respectively; LPC: lysophosphatidylcholine; DHA: docosahexaenoic acid; F.A.: fatty acid; S.D.: standard deviation.
